# Comparison of fecal and oral collection methods for studies of the human microbiota in two Iranian cohorts

**DOI:** 10.1186/s12866-021-02387-9

**Published:** 2021-11-22

**Authors:** Zeni Wu, Autumn G. Hullings, Reza Ghanbari, Arash Etemadi, Yunhu Wan, Bin Zhu, Hossein Poustchi, Behnam Bagheri Fahraji, Mohammad Javad Zare Sakhvidi, Jianxin Shi, Rob Knight, Reza Malekzadeh, Rashmi Sinha, Emily Vogtmann

**Affiliations:** 1 Metabolic Epidemiology Branch, Division of Cancer Epidemiology & Genetics, National Cancer Institute, National Institutes of Health, Bethesda, MD USA; 2 Digestive Oncology Research Center, Digestive Disease Research Institute, Tehran University of Medical Science, Tehran, Iran; 3 Frederick National Laboratory for Cancer Research/Leidos Biomedical Research Laboratory, Inc., Frederick, MD USA; 4 Cancer Genomics Research Laboratory, Division of Cancer Epidemiology & Genetics, National Cancer Institute, National Institutes of Health, Bethesda, MD USA; 5 Department of Epidemiology, School of Public Health, Shahid Sadoughi University of Medical Sciences, Yazd, Iran; 6 Department of Occupational Health, School of Public Health, Shahid Sadoughi University of Medical Sciences, Yazd, Iran; 7 Biostatistics Branch, Division of Cancer Epidemiology & Genetics, National Cancer Institute, National Institutes of Health, Bethesda, MD USA; 8 Department of Pediatrics, University of California San Diego, La Jolla, CA USA; 9 Department of Computer Science & Engineering, University of California San Diego, La Jolla, CA USA

**Keywords:** Comparability, Feces, Iran, Microbiome, Saliva, Stability

## Abstract

**Background:**

To initiate fecal and oral collections in prospective cohort studies for microbial analyses, it is essential to understand how field conditions and geographic differences may impact microbial communities. This study aimed to investigate the impact of fecal and oral sample collection methods and room temperature storage on collection samples for studies of the human microbiota.

**Results:**

We collected fecal and oral samples from participants in two Iranian cohorts located in rural Yazd (*n* = 46) and urban Gonbad (*n* = 38) and investigated room temperature stability over 4 days of fecal (RNA*later* and fecal occult blood test [FOBT] cards) and comparability of fecal and oral (OMNIgene ORAL kits and Scope mouthwash) collection methods. We calculated interclass correlation coefficients (ICCs) based on 3 alpha and 4 beta diversity metrics and the relative abundance of 3 phyla. After 4 days at room temperature, fecal stability ICCs and ICCs for Scope mouthwash were generally high for all microbial metrics. Similarly, the fecal comparability ICCs for RNA*later* and FOBT cards were high, ranging from 0.63 (95% CI: 0.46, 0.75) for the relative abundance of *Firmicutes* to 0.93 (95% CI: 0.89, 0.96) for unweighted Unifrac. Comparability ICCs for OMNIgene ORAL and Scope mouthwash were lower than fecal ICCs, ranging from 0.55 (95% CI: 0.36, 0.70) for the Shannon index to 0.79 (95% CI: 0.69, 0.86) for Bray-Curtis. Overall, RNA*later*, FOBT cards and Scope mouthwash were stable up to 4 days at room temperature. Samples collected using FOBT cards were generally comparable to RNA*later* while the OMNIgene ORAL were less similar to Scope mouthwash.

**Conclusions:**

As microbiome measures for feces samples collected using RNA*later*, FOBT cards and oral samples collected using Scope mouthwash were stable over four days at room temperature, these would be most appropriate for microbial analyses in these populations. However, one collection method should be consistently since each method may induce some differences.

**Supplementary Information:**

The online version contains supplementary material available at 10.1186/s12866-021-02387-9.

## Background

Cross-sectional studies have suggested the importance of the fecal and oral microbiota in the development of multiple disease outcomes, including cancer, atherosclerosis, and obesity [1–3]. Large-scale, prospective cohort studies with sample collections for microbiota analysis prior to disease development are needed to better understand microbial-associated disease etiology. Prior to collecting samples in these studies, it is essential to validate sampling protocols based on likely field conditions [4]. Previous research has shown that multiple factors can affect microbial composition such as sample collection method, duration of sample storage at room temperature, DNA extraction protocol, PCR amplification step for the 16S ribosomal RNA gene, sequencing protocols, and bioinformatics procedures [5–12]. Other studies have investigated the impact of sample collection method and duration of sample storage on microbial community measures [13–15]. However, few studies have investigated the impact of these logistical challenges in low- and middle-income countries and in rural versus urban settings where the gold standard collection method may not be feasible (i.e., fresh, immediately frozen for fecal samples). Since there is no community gold standard method for oral microbiota due to oral community differences by site in the oral cavity, [16] other collection methods such as the OMNIgene oral collection kit and Scope mouthwash must be compared to each other.

The Golestan Cohort Study (GCS), launched in 2004, and the Prospective Epidemiological Research Studies of Iranian Adults (PERSIAN) Cohort, launched in 2014, are two population-based cohort studies investigating risk factors for chronic diseases in the Iranian population in both rural (Yazd) and urban (Gonbad) settings [17, 18]. To further investigate potential differences in the impact of fecal and oral sample collection methods on microbial communities in these populations, we collected fecal samples using RNA*later* stabilizing solution and fecal occult blood test (FOBT) cards and collected oral samples using the OMNIgene ORAL saliva collection kit and Scope mouthwash in the GCS and PERSIAN cohorts. The aim of this study was to investigate the impact of room temperature storage on the collected samples (RNA*later,* FOBT for fecal samples, and Scope mouthwash for oral samples) and compare microbial communities from fecal samples collected in RNA*later* or an FOBT card and from oral samples collected using the OMNIgene ORAL kit or Scope mouthwash. Results showed that RNA*later*, FOBT cards and Scope mouthwash were stable up to 4 days at room temperature and that fecal samples collected using RNA*later* and FOBT cards were generally similar while oral samples collected using the OMNIgene oral kit and a Scope mouthwash were somewhat distinct.

## Results

### Population characteristics and sample collection

Fifty participants (25 male and 25 female) were randomly invited in Gonbad (GCS, rural area) and Yazd (PERSIAN cohort, urban area), respectively. A total of 84 individuals agreed to participate including 38 participants (76%) from Gonbad and 46 participants (92%) from Yazd. Fecal samples were collected either at the clinic or at home using a Sarstedt tube with RNA*later* stabilizing solution and FOBT cards. Oral samples were collected at a clinic visit using the OMNIgene oral collection kit and Scope mouthwash. At least one aliquot of each sample type was frozen immediately at − 80 °C (day-0) and one aliquot of the RNA*later*, FOBT cards, and Scope mouthwash samples was frozen at − 80 °C after sitting at room temperature for 4 days (day-4). Since OMNIgene ORAL samples are advertised to be stable at room temperature for up to 3 weeks, no day-4 aliquots were created.

Descriptive characteristics of the selected participants and the numbers of fecal and oral samples collected are presented in Table 1. Participants from Gonbad were predominantly female (57.89%) with a mean age of 53.08 years (standard deviation [SD] 1.78). Yazd participants were mostly male (60.87%) with a mean age of 45.24 years (SD 10.54).

**Table 1:** Demographics of study participants and collection methods for fecal and oral samples collected for microbiome analysis in Gonbad and Yazd, Iran

**Characteristic**	**Gonbad (N participants = 38)**		**Yazd (N participants = 46)**	
**Age in years (mean, std)**	53.08 (1.78)		45.24 (10.54)	
**Sex (n, %)**				
Male	16 (42.11)		28 (60.87)	
Female	22 (57.89)		17 (36.96)	
Missing	0 (0.00)		1 (2.17)	
**Oral Sample Collection Method**	**Day-0 (n samples)**	**Day-4 (n samples)**	**Day-0 (n samples)**	**Day-4 (n samples)**
OMNIgene	37	0	40	0
Scope mouthwash	33	28	37	27
**Fecal Sample Collection Method**				
RNA*later*	34	31	35	33
Fecal occult blood test card	36	33	34	33

**Fig. 1:**
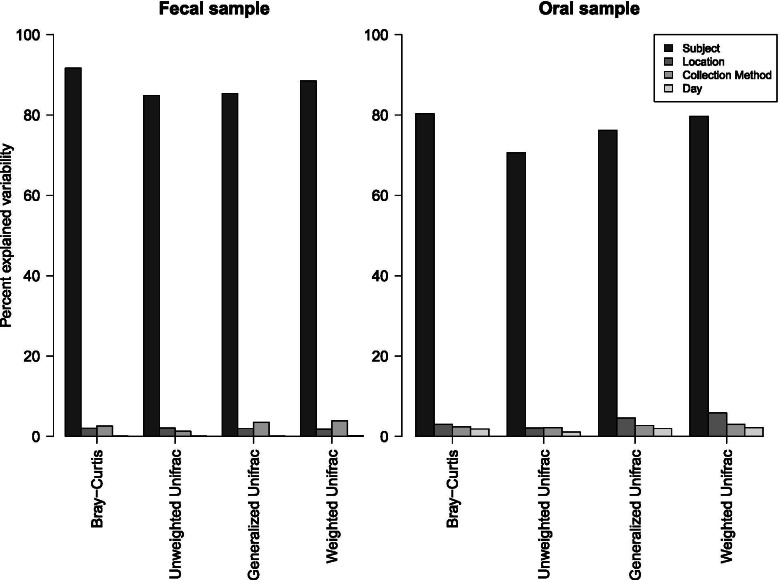
Percent microbial variability explained by subject (darkest grey), location (medium-to-dark grey), sample collection method (medium-to-light grey), and day (light grey) was calculated for beta-diversity estimates from Bray-Curtis, unweighted UniFrac, weighted UniFrac, and generalized UniFrac using a distance-based coefficient of determination (R^2^)

As shown in Fig. 1, between-subject variability explained most of the overall microbial community composition (i.e., beta diversity) for both fecal (85–92%) and oral samples (71–80%) while location, collection method, and length of storage each explained 0- < 10%. Overall, samples from Yazd had a higher relative abundance of the phylum *Bacteroidetes*, but lower amounts of *Firmicutes* in both fecal and oral samples compared to samples from Gonbad (Additional file 1 Fig. S1).

### Stability of fecal samples

**Fig. 2:**
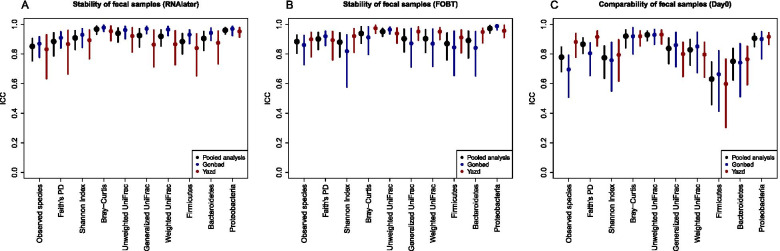
**A**: Interclass correlation coefficients (ICC) for stability using RNA*later* frozen after 4 days at room temperature (day-4) compared to immediately frozen (day-0) for alpha diversity, beta diversity metrics, and the three most dominant phyla (i.e., *Firmicutes*, *Bacteroidetes*, *Proteobacteria*) in fecal samples from Yazd and Gonbad, Iran, (N participants = 84, n samples = 269). Phylum relative abundances were square root transformed prior to calculating ICCs. *P*-values for testing statistical heterogeneity between Gonbad and Yazd are shown in Additional files 2 Table S1 and S3. **B**: ICCs for evaluating stability using fecal occult blood test (FOBT) card cards frozen after 4 days at room temperature (day-4) compared to immediately frozen (day-0). **C**: ICCs for evaluating comparability between RNA*later* and FOBT card for immediately frozen samples (day-0)

Intraclass correlation coefficients (ICCs) for stability of fecal samples collected with RNA*later* and FOBT card frozen day-0 vs. day-4 are shown in Fig. 2A-B (exact ICCs and 95% CIs are listed in Additional file 2 Table S1). Overall, the pooled stability ICCs for RNA*later* and FOBT card were all considered good to excellent (i.e., ≥0.75) for alpha and beta diversity metrics, and the most dominant phyla (i.e., *Firmicutes, Bacteroidetes, Proteobacteria*) (Fig. 2A-B). For example, the pooled ICC for observed species was 0.85 (95% CI: 0.76, 0.91) for RNA*later* and 0.88 (95% CI: 0.80, 0.93) for the FOBT card. Comparing the two study sites, stability ICCs for RNA*later* samples were higher for Gonbad compared to Yazd for all microbial metrics, and there was significant heterogeneity in the estimates for all of the beta diversity metrics and the relative abundance of *Firmicutes* and *Bacteroidetes* (Fig. 2A and Table S1). Conversely, for the FOBT cards, Gonbad generally had lower stability ICCs compared to Yazd with statistically significant heterogeneity in the estimates for the Shannon Index, Bray-Curtis, generalized Unifrac, weighted Unifrac, and the relative abundance of *Bacteroidetes* and *Proteobacteria* (Fig. 2B). Stability Spearman correlation coefficients (SCCs) for RNA*later* and FOBT cards were similar to ICCs (Additional file 1 Fig. S2A-B).

Results from the differential relative abundance analyses at phylum and genus level for stability of fecal samples are shown in Additional file 2 Table S2. After 4 days at room temperature, fecal samples collected with RNA*later* had higher levels of one phylum, *Actinobacteria* (Day-0 mean 0.0137 SD 0.0183, Day-4 mean 0.0203 SD 0.0345, false discovery rate [FDR] for Day-0 and Day-4 comparison = 0.009), and one genus, *Collinsella* (Day-0 mean 0.0023 SD 0.0038, Day-4 mean 0.0036 SD 0.0058, FDR = 0.006), but lower levels of the genus *Coprococcus 3* (Day-0 mean 0.0026 SD 0.0023, Day-4 mean 0.0019 SD 0.0021, FDR = 0.009). There were no statistically significant differences in phylum- or genus-level relative abundances for samples collected by FOBT card after 4 days at room temperature compared to immediately frozen samples.

### Comparability of fecal samples

We calculated ICCs and SCCs to evaluate comparability between collection methods (i.e., comparing RNA*later* to FOBT cards) for the day-0 samples and the comparability ICCs are shown in Fig. 2C (exact ICCs and 95% CIs are listed in Additional file 2 Table S3). In general, the ICCs were consistent for each metric. For example, the ICCs for the beta diversity matrices were all excellent (e.g., pooled ICC for unweighted Unifrac: 0.93; 95% CI: 0.89, 0.96), while the ICC for the relative abundance of *Firmicutes* was the lowest, although still moderate (pooled ICC: 0.63; 95% CI: 0.46, 0.75). There was some heterogeneity detected between the two sites for observed species and Faith’s phylogenetic diversity [PD], with Gonbad (observed species ICC: 0.70; 95% CI: 0.52, 0.80; Faith’s PD ICC: 0.81; 95% CI: 0.67, 0.88) having lower ICCs than in Yazd (observed species ICC: 0.88; 95% CI: 0.79, 0.94; Faith’s PD ICC: 0.92; 95% CI: 0.85, 0.96). SCCs were generally higher than ICCs, for both pooled and study-specific estimates and the SCC for the relative abundance of *Firmicutes* was in range with the other phyla (Additional file 1 Fig. S2 C).

Table S4 (Additional file 2) shows the differential relative abundance analyses for comparability of RNA*later* to FOBT cards. The relative abundance of many taxa were significantly different between RNA*later* and FOBT cards. For the two most abundant phyla, fecal samples collected using an FOBT card had a lower mean abundance of phylum Bacteroidetes (RNA*later* mean, 0.5198 SD 0.2103; FOBT cards mean 0.4004, SD 0.2016; FDR < 0.001) but a higher mean abundance of phylum *Firmicutes* (RNA*later* mean 0.3909, SD 0.1790; FOBT cards mean 0.5147 SD 0.1789; FDR < 0.001) compared to RNA*later*. Of the 12 genera with a mean relative abundance greater than 1% using both methods, 6 genera were higher in RNA*later* fecal samples, including *Bacteroides*, *Alloprevotella*, *Prevotella 2*, *Prevotella 9*, *Ruminococcaceae UCG-002*, and *Succinivibrio*, and 6 genera were higher in FOBT cards fecal samples, including *Bifidobacterium*, *Faecalibacterium*, *Ruminococcus 2*, *Subdoligranulum*, *[Eubacterium] coprostanoligenes group*, and an unidentified *Lachnospiraceae* genus.

### Stability of oral samples

**Fig. 3:**
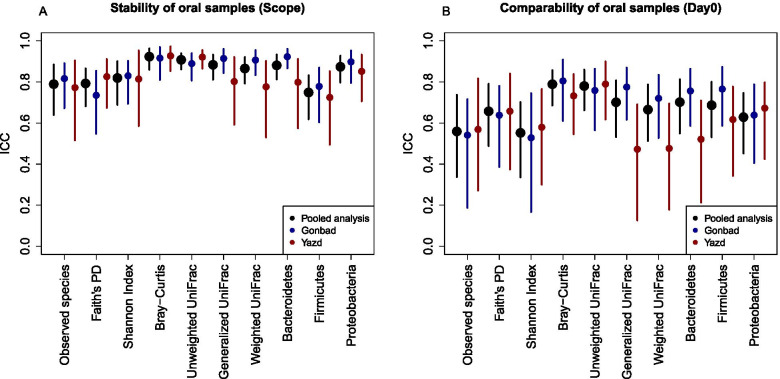
**A**: Interclass correlation coefficients (ICC) for stability using Scope mouthwash frozen after 4 days at room temperature (day-4) compared to immediately frozen (day-0) for alpha diversity, beta diversity metrics, and the three most dominant phyla (i.e., *Bacteroidetes*, *Firmicutes*, *Proteobacteria*) in oral samples from Yazd and Gonbad, Iran, (N participants = 84, n samples = 202). Phylum relative abundances were square root transformed prior to calculating ICCs. P-values for testing statistical heterogeneity between Gonbad and Yazd are shown in Additional files 2 Table S5 and S7. **B**: ICCs for evaluating comparability between OMNIgene and Scope mouthwash for immediately frozen samples (day-0)

We calculated ICCs and SCCs to evaluate stability of Scope mouthwash samples (i.e., comparing day-0 to day-4) and the stability ICCs are presented in Fig. 3A (exact ICCs and 95% CIs are listed in Additional file 2 Table S5). Pooled ICCs were all good to excellent, with ICCs ranging from 0.75 (95% CI: 0.62, 0.83) for the relative abundance of *Firmicutes* to 0.92 (95% CI: 0.86, 0.96) for Bray-Curtis. Some heterogeneity was detected between the two sites. In particular, the ICCs for generalized UniFrac (Gonbad ICC: 0.91, 95% CI: 0.84, 0.96; Yazd ICC: 0.80, 95% CI: 0.61, 0.91), weighted UniFrac (Gonbad ICC: 0.91, 95% CI: 0.84, 0.95; Yazd ICC: 0.78, 95% CI: 0.56, 0.90), and the relative abundance of *Bacteroidetes* (Gonbad ICC: 0.92, 95% CI: 0.87, 0.96; Yazd ICC: 0.80, 95% CI: 0.57, 0.91) were all lower in Yazd compared with Gonbad. The stability SCCs were generally similar to ICCs (Additional file 1 Fig. S3 A).

Results from the differential relative abundance analyses at phylum and genus level for stability of oral samples are shown in Additional file 2 Table S6. Samples collected by Scope mouthwash had a higher abundance of the phylum *Firmicutes* (Day-0 mean 0.3354, SD 0.1157; Day-4 mean 0.3941, SD 0.1180; FDR < 0.001) but lower abundance of *Proteobacteria* (Day-0 mean 0.1203, SD 0.0911; Day-4 mean 0.0928, SD 0.0745; FDR < 0.001) after 4 days at room temperature. Of the 18 genera that varied significantly (i.e., FDR < 0.01) in Scope mouthwash samples, 10 genera increased in relative abundance including *Bifidobacterium*, *F0332*, *Streptococcus*, *Peptococcus*, *Ruminococcaceae UCG-014*, *Solobacterium*, *Selenomonas 3*, *Lautropia*, *Pseudomonas*, and an unidentified *Veillonellaceae* genus; 8 genera decreased in relative abundance including *Rothia*, *Porphyromonas*, *Alloprevotella*, *Prevotella 7*, *Gemella*, *Neisseria*, *Aggregatibacter*, and *Haemophilus* after 4 days at room temperature.

### Comparability of oral samples

We calculated ICCs and SCCs to evaluate the comparability between OMNIgene ORAL and the Scope mouthwash samples. Comparability ICCs for the oral samples were lower than those seen for the fecal samples (Fig. 3B; exact ICCs and 95% CIs are listed in Additional file 2 Table S7). The pooled ICC estimates ranged from moderate to excellent with a pooled ICC for the Shannon index of 0.55 (95% CI: 0.36, 0.70) and a pooled ICC for Bray-Curtis of 0.79 (95% CI: 0.69, 0.86). Again, some heterogeneity was detected between the two sites, with generally lower comparability for the samples from Yazd. For example, the ICC for generalized Unifrac was 0.78 (95% CI: 0.61, 0.87) in Gonbad compared to 0.47 (95% CI: 0.13, 0.69) in Yazd (p-heterogeneity = 0.002). Comparability SCCs were similar to ICCs (Additional file 1 Fig. S3 B).

Table S8 (Additional file 2) shows the differential relative abundance analyses for comparability of OMNIgene ORAL to Scope mouthwash. For the two most abundant phyla, Scope mouthwash samples had higher mean relative abundance of the phyla *Bacteroidetes* (OMNIgene mean 0.3587, SD 0.1459; Scope mean 0.4097, SD 0.1752; FDR = 0.002) and *Spirochaetes* (OMNIgene mean 0.0029, SD 0.0065; Scope mean 0.0056, SD 0.0123; FDR = 0.005)*,* but lower mean abundance of *Proteobacteria* (OMNIgene mean 0.1968, SD 0.1347; Scope mean 0.1438, SD 0.1222; FDR < 0.001) compared with the OMNIgene ORAL samples. Of the 6 genera with a mean relative abundance greater than 1% using both methods, 5 genera had a higher abundance in OMNIgene samples, including *Rothia*, *Alloprevotella*, *Fusobacterium*, *Haemophilus*, whereas genus *Prevotella 6* had a higher relative abundance in Scope mouthwash samples.

## Discussion

In this study, we investigated the stability and concordance of two fecal collection methods (RNA*later* stabilizing solution and FOBT card) and two oral collection methods (the OMNIgene ORAL kit and Scope mouthwash). When looking at overall community variability, the observed variation in both fecal and oral microbial communities was primarily explained by inter-individual differences with little microbial variability related to geographic location, collection method, or freezing timepoint. Looking comprehensively at metrics of alpha diversity, beta diversity, and the relative abundance of three specific phyla, we found that RNA*later*, FOBT card, and Scope mouthwash were stable at room temperature for up to 4 days. In addition, samples preserved in RNA*later* had similar microbial community characteristics to samples collected using an FOBT card, but differences in the relative abundance of some phyla and genera were observed. OMNIgene ORAL samples were less similar to the Scope mouthwash samples, although the oral samples still had moderate to excellent comparability.

Previous studies have investigated the stability and comparability of fecal and oral microbiota samples using 16S rRNA gene sequencing and shotgun sequencing [5, 8, 10, 19–24]. For fecal samples after 4 days at room temperature, there were no substantial differences in relative abundance for samples collected by FOBT card [5, 8]. Similar to our results, FOBT and the comparable Flinders Technology Associates (FTA) cards for fecal collection samples were relatively stable and tended to perform well for 3–5 days and up to 8 weeks at room temperature [5, 8, 10, 19] with stable relative abundances for phylum *Firmicutes*, *Actinobacteria*, and *Bacteroidetes* [8, 10]. RNA*later* samples in our study were fairly stable, with a few differentially abundant taxa in the phylum *Actinobacteria* and the *Collinsella* and *Coprococcus 3* genera after room temperature storage for 4 days*.* RNA*later* has been investigated in several previous studies with mixed results [5, 8–10, 19, 25–28]. Compared to immediately frozen samples, some studies reported no change in relative abundance of taxa [5, 8, 28] or diversity [5, 9, 19, 28] after 4 and 7 days at room temperature. Other studies saw either an increase [9] or decrease [8] in Shannon Index after 3 and 7 days at room temperature. Furthermore, some studies reported either an increase [19] or decrease [25] in *Firmicutes* and either an increase [25] or decrease [19] in *Bacteroidetes* after 3 and 7 days at room temperature.

When we compared the fecal samples collected by RNA*later* and the FOBT card, most of the microbial metrics did not differ. Similar to our results, other studies found no major differences in alpha or beta diversity metrics between FOBT cards and RNA*later* [8, 14]. While one study found similar relative abundances across common phyla [8], our study saw fecal samples collected by FOBT card had a significantly lower abundance of *Bacteroidetes* but an higher abundance of two phyla, *Actinobacteria* and *Firmicutes* (FDR < 0.001) compared to RNA*later* samples. In addition, we found relative abundance differences for a number of genera including *Bacteroides* and *Bifidobacterium*, but it is not possible to determine whether these were increases or decreases in these taxa (or possibly changes in other taxa) due to the relative nature of these abundances.

Our results suggest that samples collected by Scope mouthwash are generally stable for most microbial metrics, but there was higher abundance of the phylum *Firmicutes* and lower *Proteobacteria* after 4 days at room temperature. Similar to our results, samples collected with mouthwash in previous studies found microbial composition was stable for 4 days and 1–2 weeks variable lengths of time [7, 22, 23]. One study also found an increase in relative abundance of *Firmicutes* and a decrease in *Bacteroidetes, Proteobacteria,* and *Fusobacteria* after 4 days at room temperature with Scope mouthwash samples [7]. Although we did not test the OMNIgene ORAL samples for stability, at least one previous study on oral collection methods found that the OMNIgene ORAL kit had similar bacterial diversity after 2–7 days of storage at room temperature [20].

Previous studies detected differences between samples collected by Scope mouthwash compared to OMNIgene kit, but between-subject variability tended to outweigh collection method differences [7, 24]. In our study, ICCs comparing the OMNIgene ORAL kit to Scope mouthwash were generally the lowest of the ICCs in this study, although they were never in the poor ICC range (< 50%). Similarly, one study found comparability ICCs between Scope mouthwash and OMNIgene ORAL kit were also relatively low with the exception of the relative abundance of *Bacteroidetes* and observed species [7]. Compared to OMNIgene samples, our Scope mouthwash samples also had significant differences in the relative abundance of specific phyla, including a higher abundance of *Bacteroidetes* and *Spirochaetes* and lower abundance of *Proteobacteria*. One study also found lower levels of *Proteobacteria* in Scope mouthwash, but in contrast to our findings, found a higher relative abundance of *Spirochaetes* in Scope mouthwash samples compared to OMNIgene samples [7]. A number of relative abundance differences were also observed at the genus level.

Our study found some differences in the stability or comparability of fecal and oral samples by location. For example, stability ICCs for RNA*later* samples were slightly lower in Yazd samples but ICCs for FOBT cards were lower in Gonbad samples. It is unclear why the stability may differ by location or rurality, and was dependent on the collection method, but these differences highlight the importance of conducting a pilot study to optimize the best method for collecting fecal and oral collection before conducting a large prospective cohort. Previous studies have found that the gut microbiota composition differs by geographic location [13, 15], but it is possible that these differences could be partially related to handling characteristics such as temperature, collection methods, or processing procedures.

This study had several limitations. First, since we did not create day-4 samples for the OMNIgene ORAL specimens, we were unable to assess stability for these samples. Another limitation is that we only assessed stability and comparability using 16S rRNA gene sequencing. It is unclear whether estimates of stability or comparability would differ using microbial metrics derived from whole-genome shotgun metagenomic sequencing and we were unable to evaluate the impact on room temperature storage and collection method on microbial functionality. Furthermore, this study only assessed stability at room temperature for 4 days, which may not cover all shipping and storage conditions for field studies.

This study also had many strengths. Most previous collection comparison studies were conducted in developed countries, with few conducted in low-to-middle income countries [29]. This study included individuals from 2 provinces within Iran from both rural and urban settings and demonstrated the acceptability of collecting fecal and oral samples in these populations. In addition, this study demonstrated the utility of collecting FOBT card for fecal and Scope mouthwash for oral microbiota assessment, which are both cost-effective for large-scale cohorts.

## Conclusions

In conclusion, results from this methods study helps to inform larger cohort studies of best methodological practices when collecting fecal and oral microbial samples, particularly in the low- and middle-income countries. Similar to previous methods studies of microbial samples, our study concluded that both RNA*later* stabilizing solution and the FOBT card are acceptable to collect and preserve fecal samples and both the OMNIgene ORAL and Scope mouthwash are acceptable to collect and preserve oral samples. However, due to differences observed for various collection methods in microbial structure it is advisable to use one method for each type of microbial sample for comparisons when scaling to larger studies. Future studies should investigate potential heterogeneity in stability and comparability between populations and investigate factors associated with this heterogeneity.

## Methods

### Study population

A summary of the population characteristics, collection methods, and number of microbial samples used for each method is outlined in Table 1 and in the participant study flowchart (Additional file 1 Fig. S4). The GCS and PERSIAN cohorts were described previously [17, 18]. In brief, the GCS consists of 50,045 participants aged 40–75 years, who were sampled from the urban area of Gonbad City (*n* = 10,032) and surrounding rural areas (*n* = 40,013) from January 2004–June 2008 [18]. The PERSIAN cohort has accrued approximately 165,000 participants since 2014 and is still accruing participants with the aim to include 180,000 Iranians aged 35–71 years from 18 geographic areas in Iran [17]. For the GCS, the inclusion criteria were comprised of not having a current or previous diagnosis of upper gastrointestinal cancer and not being a temporary resident of the area. For the PERSIAN Cohort, the inclusion criteria included being of Iranian descent, living in the study area for at least 6 months, and not having any physical or psychological disability that would prevent them from completing the enrollment process. 50 participants (25 male and 25 female) were randomly selected from Gonbad for GCS and Yazd for the PERSIAN cohort to participate in a pilot study to measure their fecal and oral microbiota. The final analytic cohort consisted of 84 individuals with a participation rate of 84%. Possible reasons for not completing the sample collection ranged from worry about the sampling (*n* = 9), inability to complete the sample procedures (*n* = 0), not having enough time (n = 1), or unknown (*n* = 6). Basic demographic information such as age and sex were collected via questionnaire at the time of sample collection. The GCS was approved by the ethical review committees of the Digestive Disease Research Center of Tehran University Medical Science, the International Agency for Research on Cancer, and the United States National Cancer Institute. The PERSIAN Cohort received approval from the ethics committees of the Ministry of Health and Medical Education, the Digestive Disease Research Institute (Tehran University of Medical Sciences), and each participating university.

### Fecal sample collection

Participants received a fecal collection kit containing a stool collection container, two Sarstedt feces tubes (Numbrect, Germany) filled with 2.5 mL of RNA*later* stabilizing solution (Ambion, Inc., Austin, Texas), two FOBT cards (Hemoccult II Elite Dispensapak Plus, Beckman Coulter, Brea, California), and a wooden applicator stick. After the feces were collected in the container, the participants were instructed to add a scoop of the feces into each of the two Sarstedt feces tubes and spread a small amount of feces onto the two FOBT cards. Approximately half of the participants provided a fecal sample at the clinic and half collected their samples at home. For participants collecting fecal samples in the clinic, one Sarstedt tube and one FOBT card were immediately returned to the laboratory and frozen at − 80 °C (day-0). For those collected at home, samples were delivered to the laboratory within a few hours after collection and then one Sarstedt tube and one FOBT card were frozen at − 80 °C (day-0). Whether collected on site or at home, each participants’ second Sarstedt tube and FOBT card were left room temperature for 96 h (day-4) before being frozen at -80 °C.

### Oral sample collection

At the clinic visit, participants provided oral samples using the OMNIgene ORAL OM-505 saliva collection kit (DNAGenotek, Ontario, Canada) and Scope mouthwash (Proctor & Gamble). Participants were asked to refrain from eating and smoking at least 20 min prior to sample collection. All OMNIgene samples were collected before mouthwash samples by spitting into a pre-labeled tube until the amount of saliva reached the “fill line”. The cap was closed to release the preservative solution and mixed. Next, the participant used 10 mL of Scope mouthwash to swish for 5 s and gargle for 5 s three times each for a total of 30 s before spitting into a pre-labeled collection cup. Two 1.8 mL aliquots were created from the Scope mouthwash samples. The first was frozen immediately at -80 °C while second aliquot was kept at room temperature for 96 h (day-4) and then stored at -80 °C. After collection, OMNIgene tubes were processed according to DNAGenotek’s aliquoting protocol (https://www.dnagenotek.com/us/pdf/PD-PR-00214.pdf). Briefly, the OMNIgene funnel cap was replaced with the standard cap and then the tube was vigorously shaken for 8 s or longer by hand. Prior to aliquoting and freezing, the sample was incubated at 50 °C for 1 h in a water bath or for 2 h in an air incubator. After incubation, two 1.0 mL aliquots were frozen immediately at -80 °C (day-0).

### DNA extraction and sequencing

After storage in Tehran for approximately 6 months, all fecal and oral samples were shipped to the NCI repository on dry ice and then to the Knight lab at the University of California San Diego, California. Samples were kept at 4 °C while plating. Swabs were used to sample stool specimens for DNA extraction (Puritan Cotton Tipped Applicators – Puritan Medical Products). Out of the total 6 DNA extraction batches, 5 batches contained 8 blank quality control (QC) samples, and 1 batch contained 23 blank QC samples (*n* = 63 total QC samples). DNA extraction, PCR amplification, and 16S rRNA amplicon were performed using the Earth Microbiome Project protocols (http://www.earthmicrobiome.org/protocols-and-standards/16s). In brief, DNA extraction was performed using the Qiagen MagAttract PowerSoil DNA kit as described previously [30]. Amplicon PCR amplification was performed on the V4 region of the 16S rRNA gene using the primer pair 515f to 806r with Golay error-correcting barcodes on the reverse primer (FWD:GTGCCAGCMGCCGCGGTAA; REV:GGACTACHVGGGTWTCTAAT). Amplicons were barcoded, pooled in equal concentrations for sequencing, purified with the Qiagen UltraClean PCR cleanup kit, and 2 × 250 bp paired end sequencing performed on the Illumina MiSeq sequencing platform.

### Bioinformatic processing

Default settings were used for all steps in the bioinformatic processing unless otherwise mentioned. Sequence data were demultiplexed and minimally quality filtered using the QIIME 1.9.1 script split_libraries_fastq.py, with a Phred quality threshold of 3 and default parameters to generate per-study FASTA sequence files. The average number of RAW sequencing reads was 17,777 reads/sample. After filtering, merging paired-end reads, and removing chimeras, there was an average of 14,907 reads/sample. A total of 4077 unique ASVs were identified. The average number of detected reads for blank samples were very low (67% of samples had < 100 reads, 28% had 200–700 reads, 4% had > 10,000 reads). Sequence variants were generated by the DADA2 plugin in QIIME2 [31, 32] and taxonomy was assigned using SILVA classifier version v132, [33] with a total of 4077 ASV identified. Taxonomies were then filtered to only include bacterial sequences the phylogenic tree was generated based on all of the identified ASVs using the “DECIPHER” and “phangorn” packages in R. Based on rarefaction curves for alpha diversity (Additional file 1 Fig. S5), we rarefied each sample to 10,000 sequences per fecal sample and 5000 sequences per oral sample with 269 fecal samples and 202 oral samples used for statistical analyses. Fifteen fecal samples and 27 oral samples were excluded due to less than 10,000 sequences per fecal sample or 5000 sequences per oral sample. Alpha diversity measures (i.e., observed species, Shannon Diversity Index, and Faith’s PD) were calculated based on the alpha_diversity function in QIIME version 1.9.1. Beta diversity measures (i.e., Bray–Curtis distance, weighted Unifrac, unweighted Unifrac, and generalized Unifrac) were calculated based on the beta_diversity function in QIIME version 1.9.1.

### Statistical analysis

Statistical analyses were conducted using R, version 3.6.1. To identify potential outliers and sampling mislabeling/misclassification, we created the principal coordinates analysis (PCoA) plots based on unweighted the UniFrac distance matrix with all samples at a rarefaction value of 6000. Fecal samples that clustered with the oral samples or vice versa were identified as suspicious. In the outlier analysis, we found no evidence of mislabeling or contamination and all samples clustered by body site as expected (Additional file 1 Fig. S6).

We calculated a distance-based coefficient of determination (R^2^) from Bray-Curtis, generalized UniFrac, unweighted UniFrac, and weighted UniFrac beta diversity estimates to quantify the percentage of microbial variability explained by subject, location, collection method and length of storage using the Adonis function in the vegan package in R [34].

We calculated intraclass correlation coefficients (ICCs) to evaluate stability (freezing at day-0 vs. day-4) for a given collection method (e.g., RNA*later* and FOBT for gut microbiome, OMNIgene mouthwash vs. Scope mouthwash for oral microbiome). For each analysis, ICC was defined as the ratio ${\sigma}_b^2/\left({\sigma}_b^2+{\sigma}_w^2\right)$, where ${\sigma}_b^2$ and ${\sigma}_w^2$ were between- and within-subject variances estimated based on the “lmer” function in lme4 package [35]. Here, ${\sigma}_w^2$ measures the instability for microbiome samples freezing at day-0 vs. day-4. We calculated ICCs for three alpha diversity metrics (i.e., observed species, Faith’s PD and Shannon index), the top principal coordinate vector from 4 beta diversity metrics (i.e., Bray-Curtis, generalized UniFrac, unweighted UniFrac and weighted UniFrac distance), and the relative abundances (square root transformed) of the three most dominant phyla (i.e., *Bacteroidetes*, *Firmicutes*, and *Proteobacteria*). The top principal coordinate vector explained 28.51, 30.98, 22.08, and 56.97% of the variability among fecal samples for Bray-Curtis, generalized Unifrac, unweighted Unifrac, and weighted Unifrac, respectively. The top principal coordinate vector explained 28.76, 36.00, 25.02, and 57.91% of the variability among oral samples for Bray-Curtis, generalized UniFrac, unweighted UniFrac, and weighted UniFrac, respectively. We calculated 95% confidence intervals (CIs) based on the boot-strap technique to resample the population with replacement 1000 iterations. In addition to the pooled ICC and SCC estimates (i.e., combined data from Gonbad and Yazd for ICC and SCC calculation, referred to as the pooled analysis), we calculated estimates stratified by geographical location (i.e., Gonbad and Yazd). The *p*-values for heterogeneity between Gonbad and Yazd were calculated using the “metacor” function in the “meta” package. We also calculated Spearman correlation coefficients (SCCs) to determine whether the rank order of samples was similar between the collection methods or days at room temperature. We interpreted ICC/SCC values of < 50% as poor, 50- < 75% as moderate, 75- < 90% as good, and ≥ 90% as excellent [36].

Similarly, we evaluated consistency between two collection methods (RNA*later* vs. FOBT for gut microbiome, OMNIgene mouthwash vs. Scope mouthwash fro oral microbiome) using samples immediately frozen (day-0). For each of the analysis, ${\sigma}_w^2$ measures the variability for microbiome samples collected using two different methods frozen at day-0.

We conducted a differential abundance analysis using the Wilcoxon signed-rank test at the phylum and genus level for different collection methods and room temperature storage. Taxa present in less than 10% in all samples or relative abundance less than 0.2% were excluded from testing, similar to previous methods [7]. We corrected for multiple testing using the Benjamini-Hochberg procedure to control the false discovery rate (FDR).

## Supplementary Information

**Additional file 1.**

**Additional file 2.**

## Data Availability

The datasets generated and/or analyzed during the current study are available in the Sequence Read Archive (NCBI SRA) under BioProject ID PRJNA738943 (http://www.ncbi.nlm.nih.gov/bioproject/738943).

## Abbreviations

CI: Confidence Intervals
Faith’s PD: Faith’s Phylogenetic Diversity
FDR: False Discovery Rate
FOBT: Fecal Occult Blood Test
FTA: Flinders Technology Associates
GCS: Golestan Cohort Study
ICC: Interclass Correlation Coefficient
PCoA: Principal Coordinates Analysis
PERSIAN: Prospective Epidemiological Research Studies of Iranian Adults
QC: Quality Control
SCC: Spearman Correlation Coefficient
SD: Standard Deviation
