# Supplementary material for: Comparison of fecal and oral collection methods for studies of the human microbiota in two Iranian cohorts
Source: BMC Microbiol. 2021 Nov 22;21:324. doi: 10.1186/s12866-021-02387-9 (PMC8607576; doi:10.1186/s12866-021-02387-9)
Supplement: Supplementary file 1 — Additional file 1. [file 12866_2021_2387_MOESM1_ESM.pdf]

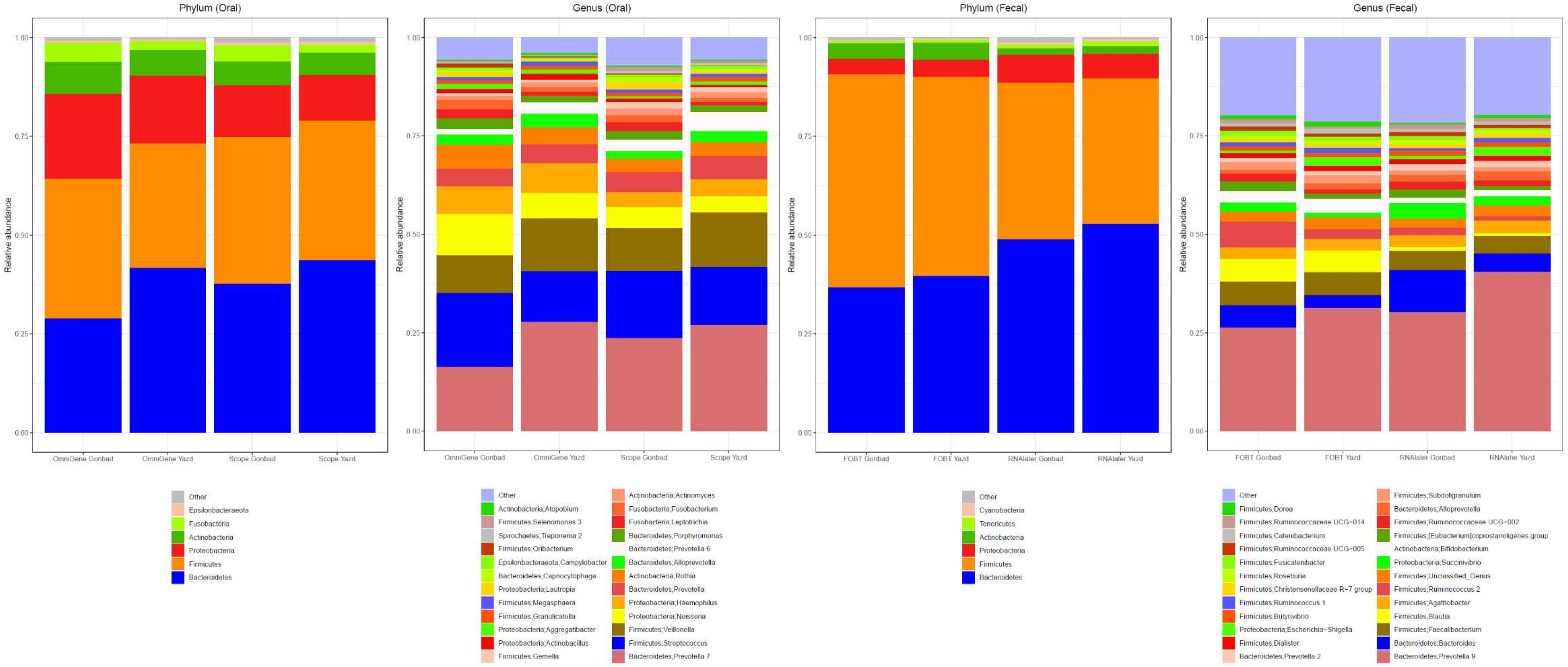

Figure S1 Relative abundance of the top 6 phyla and the top 25 genera in fecal (n samples=269) and oral samples (n samples=202) collected by FOBT card, RNA*later,* OMNIGene ORAL kit, and Scope mouthwash frozen day-0 or day-4 from Yazd and Gonbad, Iran. Abbreviations: FOBT card, fecal occult blood test cards.

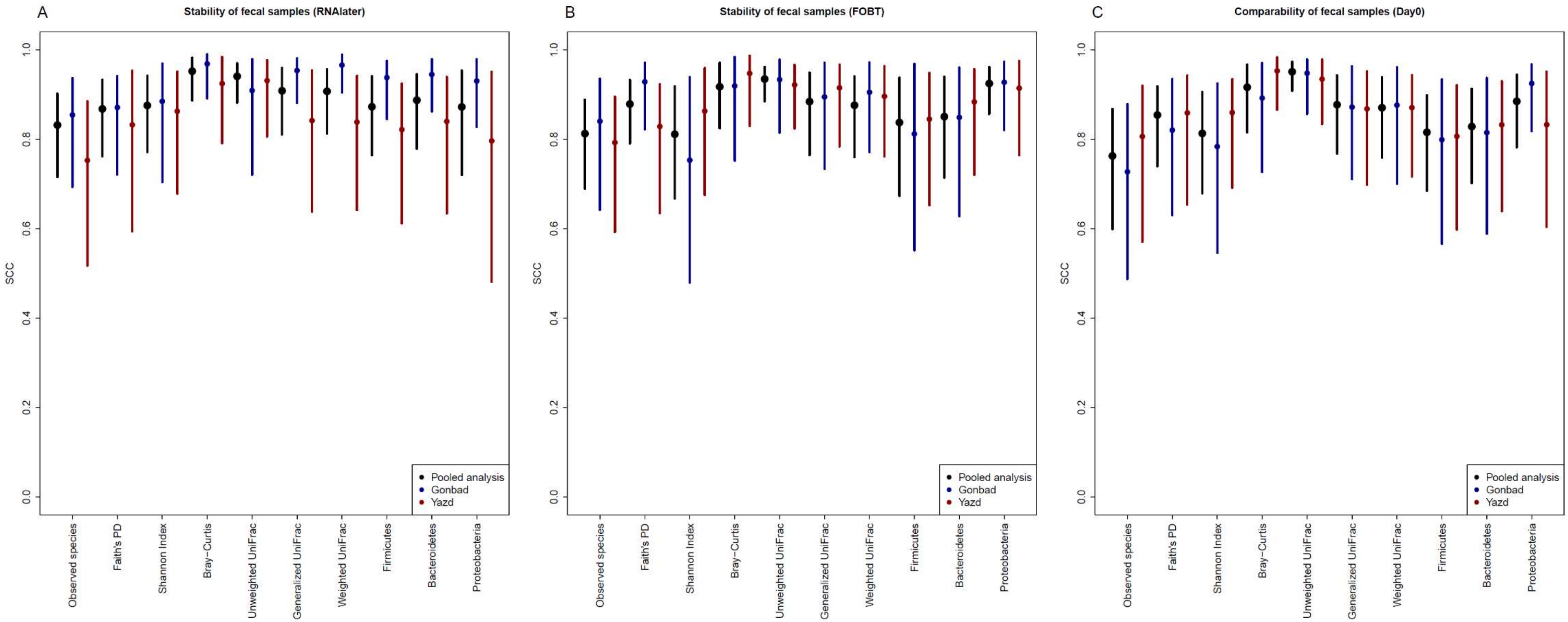

Figure S2 SCCs for stability and comparability of microbiome for alpha diversity, beta diversity metrics, and the three most dominant phyla (i.e., *Firmicutes*, *Bacteroidetes*, *Proteobacteria*) in fecal samples from Yazd and Gonbad, Iran, (N participants=84, n samples=269); phylum relative abundances were square root transformed prior to calculating SCCs. Abbreviations: SCC, Spearman correlation coefficient; FOBT, fecal occult blood test card.

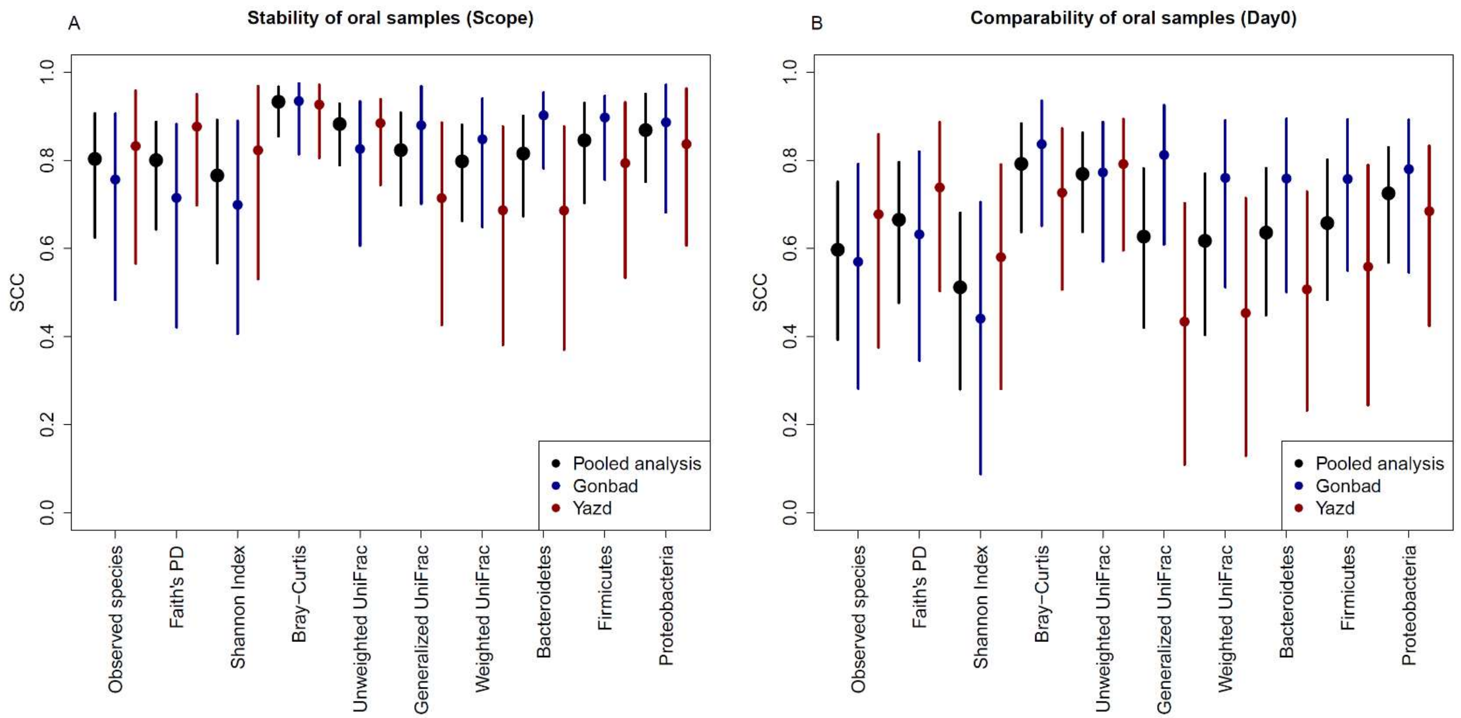

Figure S3 SCCs for stability and comparability of microbiome for alpha diversity, beta diversity metrics, and the three most dominant phyla (i.e., *Firmicutes*, *Bacteroidetes*, *Proteobacteria*) in oral samples from Yazd and Gonbad, Iran, (N participants=84, n samples=202); phylum relative abundances were square root transformed prior to calculating SCCs. Abbreviations: SCC, Spearman correlation coefficient; FOBT, fecal occult blood test card.

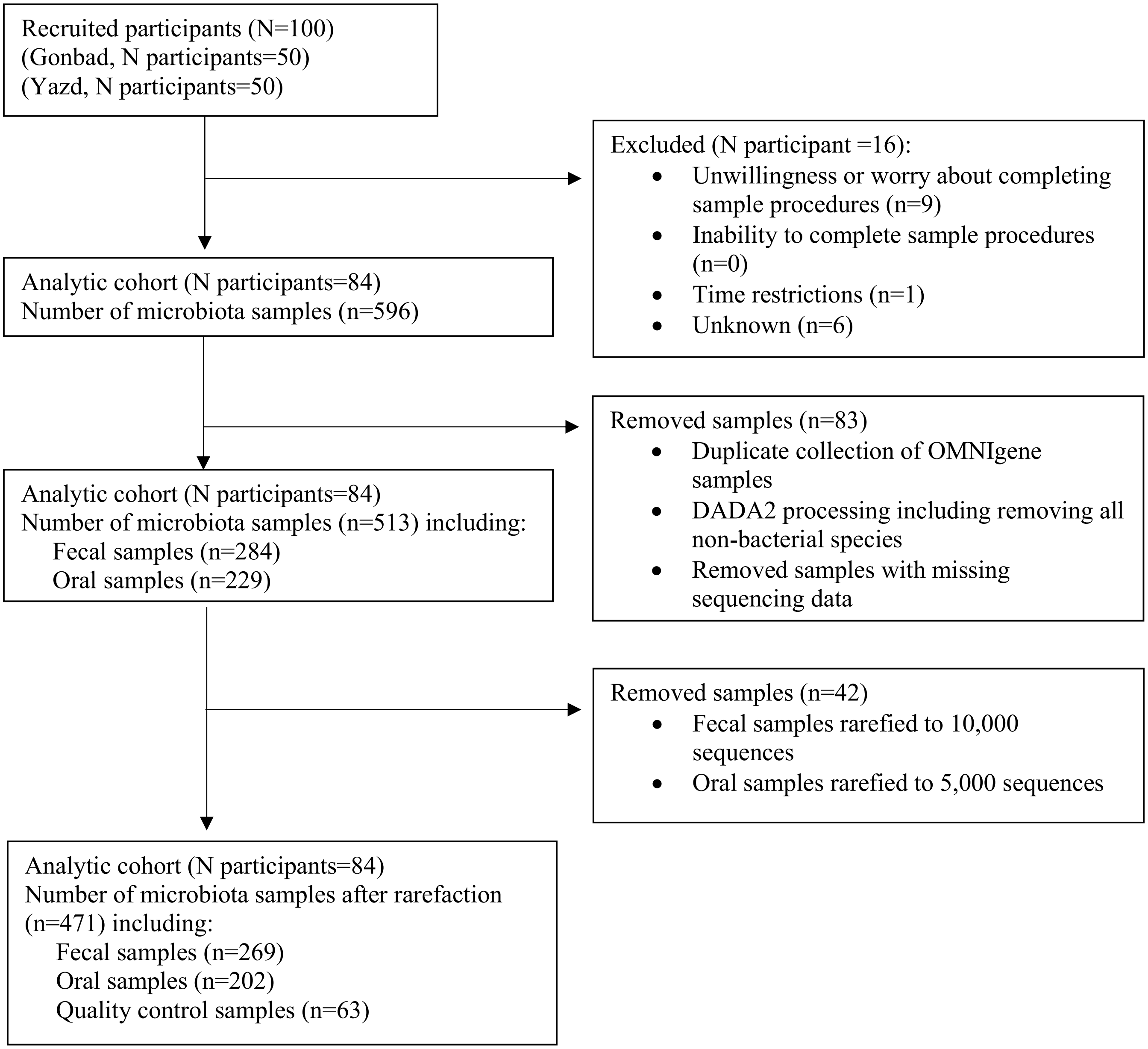

Figure S4 Participant flow chart

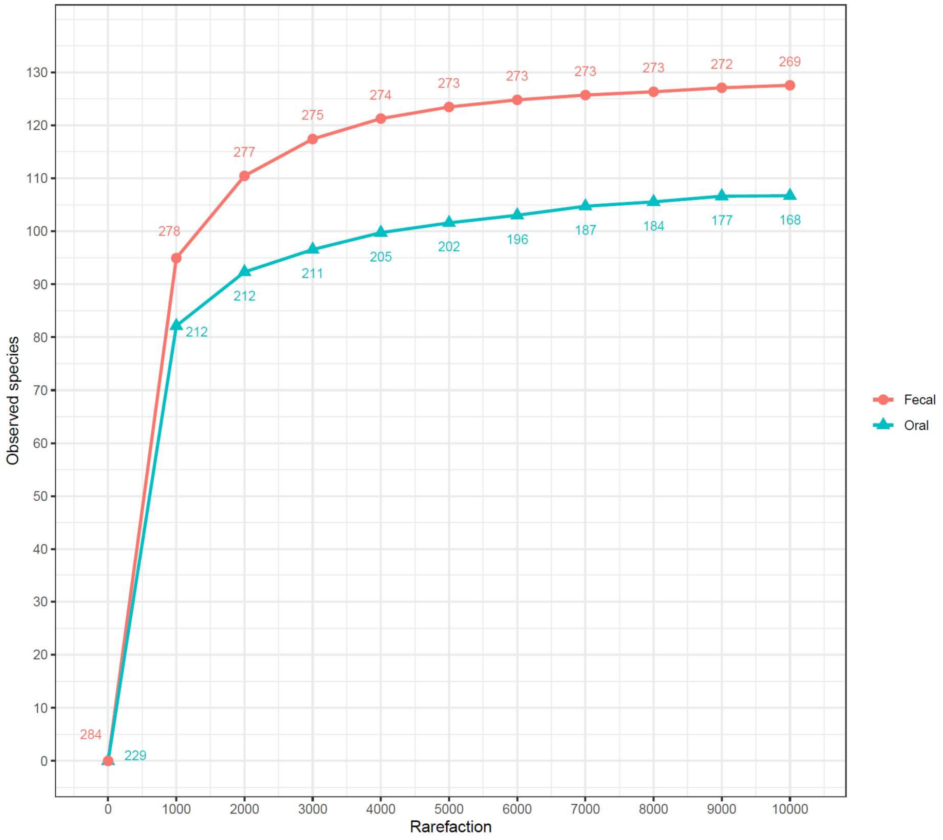

Figure S5 Rarefaction curves for observed species for fecal and oral samples. Each number indicates the number of samples at that specific rarefaction.

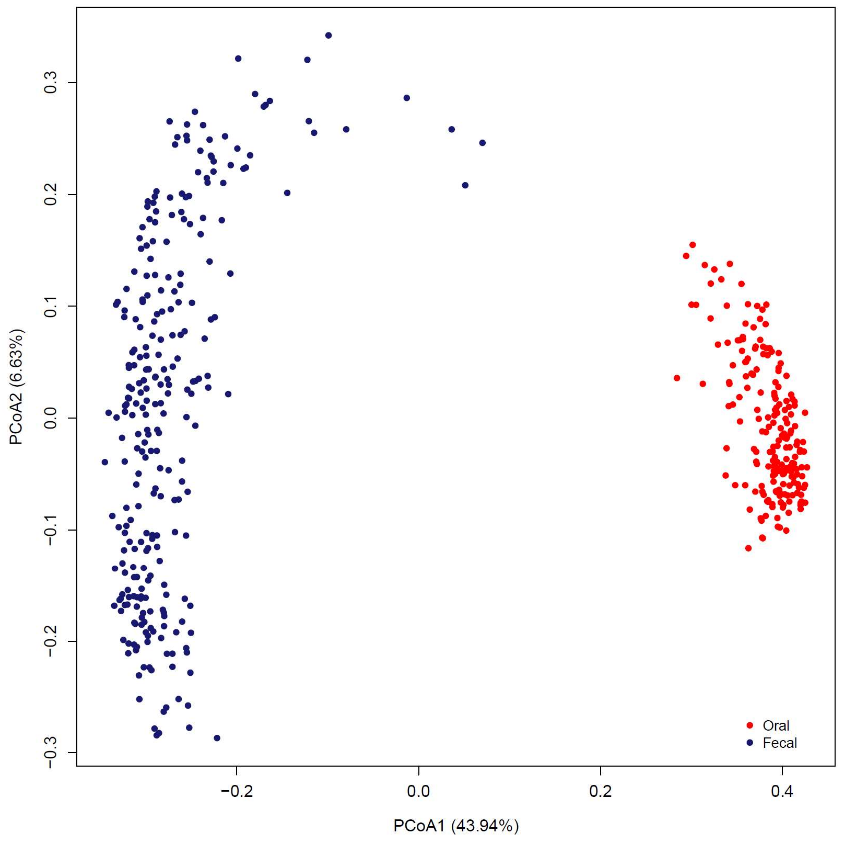

Figure S6 Principal coordinate analysis (PCoA) of the first two principal components of fecal and oral samples from Yazd and Gonbad, Iran, (n total samples=471).
